# Flat feet, prone feet, posture and dependency between them in first grade children

**DOI:** 10.1186/1748-7161-9-S1-O16

**Published:** 2014-12-04

**Authors:** Darina Zaharieva

**Affiliations:** 1 National Sports Academy, Sofia, Bulgaria

## Background

Posture is simply the position our bodies adopt in response to the effects of gravity. It is the way we hold ourselves, in sitting, standing or even lying down. Correct posture gives not only a smart appearance but also helps to prevent injury and illness of the spine. The children’s ankle-foot complex pass through various stages of development. The presence of abnormalities in them would be a logical prerequisite for the development of abnormalities in other parts of the children’s body.

## Aim

To establish frequency of flat feet, prone feet and posture and dependency between them in first grade children. The outcome of this assessment will be used to determine the choice of exercises that may improve the deficits discovered during the assessment.

## Methods

31 girls and 27 boys mean age 7 years old were studied in October and November 2012. For the purposes of that study we used: posture assessment, ankle-foot complex assessment, pedobarographi to evaluate the transverse and longitudinal arch of the foot. The plantar pressure distribution was recorded using I-Step foot scanner in erect standing position for 30 second on the foot scanning plate. The result analyzed using the Bravais-Pearson's correlation coefficient (R).

## Results

Abnormal posture were observed in 79% of children, while 12% of assess children have no deviation in ankle-foot complex. There is no significant correlation between sex and pronation (R -0.13), age and posture abnormality (R 0.08), age and pronation (R 0.14), age and flat feet (R 0.24). There is no direct correlation between flat feet and posture abnormality, as in our study there is only one such case. There is a strong correlation between pronation and postural deviations (R 0.86).

## Conclusion

The pronation in ankle-foot complex is the leading factor for the variation in the children’s posture and it should be monitored and treated during children's development. To determine the proper treatment is important to invent and use the precision assessments that separate the two deformities (pronation and low arch – flat feet).

The equipment was supported by Hodileko.bg

